# Concomitant Pulmonary Tuberculosis in Hospitalized Healthcare-Associated Pneumonia in a Tuberculosis Endemic Area: A Multi-center Retrospective Study

**DOI:** 10.1371/journal.pone.0036832

**Published:** 2012-05-22

**Authors:** Jia-Yih Feng, Wen-Feng Fang, Chieh-Liang Wu, Chong-Jen Yu, Meng-Chih Lin, Shih-Chi Ku, Yu-Chun Chen, Chang-Wen Chen, Chih-Yen Tu, Wei-Juin Su, Kuang-Yao Yang

**Affiliations:** 1 Department of Chest Medicine, Taipei Veterans General Hospital, Taipei, Taiwan; 2 Institute of Clinical Medicine; School of Medicine, National Yang-Ming University, Taipei, Taiwan; 3 Division of Pulmonary and Critical Care Medicine and Department of Respiratory Therapy, Kaohsiung Chang Gung Memorial Hospital and Chang Gung University College of Medicine, Kaohsiung, Taiwan; 4 Department of Respiratory Care, Chang Gung University of Science and Technology, Chiayi, Taiwan; 5 Department of Internal Medicine, Chiayi Branch, Taichung Veterans General Hospital, Taichung, Taiwan; 6 Department of Respiratory Therapy, College of Health Care, China Medical University, Taichung, Taiwan; 7 Department of Internal Medicine, National Taiwan University Hospital, Taipei, Taiwan; 8 School of Medicine, National Taiwan University, Taipei, Taiwan; 9 Division of Pulmonary and Critical Care Medicine, Department of Internal Medicine, National Taiwan University Hospital, Taipei, Taiwan; 10 Department of Medical Research and Education, National Yang-Ming University Hospital, Yilan, Taiwan; 11 School of Medicine, National Yang-Ming University, Taipei, Taiwan; 12 Medical Intensive Care Unit, Department of Internal Medicine, National Cheng-Kung University Hospital, Tainan, Taiwan; 13 Division of Pulmonary and Critical Care Medicine, Department of Internal Medicine, China Medical University Hospital, Taichung, Taiwan; 14 School of Medicine, China Medical University, Taichung, Taiwan; 15 Department of Life Science, National Chung Hsing University, Taichung, Taiwan; Institut de Pharmacologie et de Biologie Structurale, France

## Abstract

**Background:**

In tuberculosis (TB) endemic areas, *Mycobacterium tuberculosis* is an important but easily misdiagnosed pathogen in community-acquired pneumonia (CAP). However, the occurrence of concomitant pulmonary tuberculosis (PTB) in hospitalized healthcare-associated pneumonia (HCAP) has never been investigated.

**Methods and Findings:**

Seven hundred and one hospitalized HCAP and 934 hospitalized CAP patients from six medical centers in Taiwan were included in this nationwide retrospective study. Concomitant PTB was defined as active PTB diagnosed within 60 days of admission due to HCAP or CAP. The predictors for concomitant PTB and the impact of PTB on the outcomes of pneumonia were investigated. Among the enrolled subjects, 21/701 (3%) of the HCAP patients and 25/934 (2.7%) of the CAP patients were documented to have concomitant PTB. In multivariate analysis, a history of previous anti-TB treatment (OR = 5.84, 95% CI: 2.29–20.37 in HCAP; OR = 3.33, 95% CI: 1.09–10.22 in CAP) and escalated pneumonia severity index (PSI) scores (OR = 1.014, 95% CI: 1.002–1.026, in HCAP; OR = 1.013, 95% CI: 1.001–1.026, in CAP) were independent predictors for concomitant PTB in both CAP and HCAP patients. Regarding treatment outcomes, HCAP patients with concomitant PTB were associated with more acute respiratory failure within 48 hours of admission (47.6% vs. 22.6%, p = 0.008), higher intensive care unit admission rate (61.9% vs. 35.7%, p = 0.014), longer hospitalization (39.6±34.1 vs. 23.7±27 days, p = 0.009), and higher in-hospital mortality (47.6% vs. 26.3%, p = 0.03) than those without concomitant PTB. Exposure to certain groups of antibiotics for the treatment of pneumonia was not associated with the occurrence of concomitant PTB.

**Conclusions:**

In HCAP patients, the occurrence of concomitant PTB is comparable with that in CAP patients and associated with higher PSI scores, more acute respiratory failure, and higher in-hospital mortality.

## Introduction

Tuberculosis (TB) is an important infectious disease associated with high mortality and morbidity. According to estimates of the World Health Organization (WHO), there were 9.4 million incident cases of TB, and 1.7 million people who died from TB worldwide in 2009 [1]. In Taiwan, the incidence and mortality of TB were 62 and 3.3 cases per 100,000 population, respectively, in 2008 [2]. Due to the non-specific symptoms and diverse radiographic presentations, it is not always easy to differentiate pulmonary tuberculosis (PTB) from bacterial pneumonia in a clinical setting [3]. In TB endemic areas, TB studies are frequently ordered in patients treated for community-acquired pneumonia (CAP). Empirical antibiotics, especially newer fluoroquinolones (FQNs), are used with caution as they may delay the diagnosis of PTB and are associated with worse outcomes [4], [5].

Healthcare-associated pneumonia (HCAP) is a relatively new category of pneumonia that was first defined in 2005 with unique microbiological characteristics and treatment outcomes [6], [7]. It represents a pulmonary infection that occurs before hospitalization in patients who had contact with or an exposure history to a healthcare environment [8]. HCAP patients are generally older with multiple comorbidities, which are also risk factors for the development of PTB. However, the occurrence of concomitant PTB in HCAP patients has never been evaluated. Unawareness of concomitant PTB in hospitalized HCAP patients may lead to a delayed diagnosis and transmission of *Mycobacterium tuberculosis* (MTB) in healthcare facilities. The main purpose of the present study was to investigate the occurrence and clinical predictors of concomitant PTB in hospitalized HCAP patients, and compare them to those of CAP patients. The relationship of exposure to antibiotics for the treatment of pneumonia to PTB occurrence, and the impact of concomitant PTB in the outcomes of pneumonia in these patients were also evaluated.

## Materials and Methods

### Ethics

This study was approved by the Institutional Review Boards of Taipei Veterans General Hospital; Kaohsiung Chang Gung Memorial Hospital; Taichung Veterans General Hospital; National Taiwan University Hospital; National Cheng-Kung University Hospital; and China Medical University Hospital. Being a retrospective study, informed consent was waived according to the regulations of the Department of Health, Taiwan.

### Patients

This was a nationwide retrospective cohort study conducted at six tertiary medical centers in Taiwan. Patients admitted to these hospitals with a final diagnosis of HCAP from 1 January 2007 to 31 December 2007 were eligible for inclusion. We also included hospitalized CAP patients from these hospitals during the same study period for comparison. HCAP and CAP were defined as in previous reports [9], [10]. Patients presenting with pneumonia diagnosed within 48 hours after hospitalization were included as HCAP if at least one of the following criteria was satisfied: (1) receiving regular dialysis at an outpatient clinic; (2) receiving radiation therapy or chemotherapy at an outpatient clinic; (3) undergoing repeated hospitalization within 90 days prior to the episode of current pneumonia; or (4) residing in a nursing home. Pneumonia patients diagnosed within 48 hours after hospitalization were included as CAP if none of the HCAP criteria were met. The diagnosis of pneumonia was confirmed by chest radiographic manifestations with the growth of microorganisms from cultures of respiratory specimens or clinical improvement after antibiotic therapy. Patients who had lung cancer with obstructive pneumonitis, inadequate data for review, or were younger than 18 years old were excluded from analysis.

### Clinical evaluation

Demographic profiles (age, sex), clinical characteristics (smoking habits and comorbidities), and usage of antibiotics were obtained from medical records. A history of previous anti-TB treatment was determined according to the registration database of the Centers for Disease Control (CDC), Taiwan, and the medical records in each study hospital. The chest radiographs on the day of admission were reviewed by the responsible pulmonologist at each hospital. The pneumonia scoring indices, including CURB65 (confusion, urea, respiratory rate, blood pressure, age 65) score and pneumonia severity index (PSI) were calculated as described in previous studies [11], [12]. The antibiotics used during hospitalization were classified as cephalosporin, penicillin, macrolide, and newer quinolone (including moxifloxacin and levofloxacin) groups. Exposure to these groups of antibiotics was defined if the antibiotics had been used within the last 7 days. The outcomes of pneumonia were evaluated by organ dysfunction within 48 hours, intensive care unit (ICU) admission rate, length of hospital stay, number of antibiotics used, long-term ventilator dependence rate, and mortality rate. Organ dysfunction was defined as per a previous study [13]. The severity of pneumonia was stratified according to the PSI scores (≤130, 131∼149, and ≥150) and the number of organ dysfunctions to compare with the occurrence of concomitant PTB. All the patients were followed until mortality or discharge after an improvement in pneumonia.

### Identification of concomitant PTB

As conventional TB culture methods generally require six to eight weeks to obtain the final results, pneumonia patients with active PTB diagnosed within 60 days of admission were defined as cases with concomitant PTB. The occurrence of PTB was determined according to the records in the CDC registration database, Taiwan. Only PTB that was proven by TB cultures or typical pathological findings from respiratory specimens were included for analysis.

### Statistical analysis

Statistical analysis was performed using SPSS version 17.0 software (SPSS, Chicago, IL, USA). Continuous variables between subgroups were compared with Mann-Whitney U or independent t tests, and categorical variables were compared using Pearson's chi-square or Fisher's exact tests. Binary logistic regression analysis with stepwise selection was performed to determine the independent variables for the occurrence of concomitant PTB, and odds ratios with their 95% confidence intervals were presented. A p value less than 0.1 in the univariate analysis was required for a variable to be entered into the multivariate model. Survival time was estimated by the Kaplan-Meier method, and the log-rank test was used to compare mortality in HCAP and CAP patients with or without concomitant PTB. Censored analysis was used because the observation time was limited by discharge from the hospitals. All tests were two-tailed, and a p value less than 0.05 was considered to be statistically significant.

## Results

### Patients characteristics

During the study period, a total of 718 HCAP patients were identified from the six study hospitals. Of these patients, seven were excluded due to lung cancer with obstructive pneumonitis, and 10 were excluded due to incomplete data for review. In total, 701 hospitalized HCAP patients were included for analysis. Among these patients, 323 (46.1%) were hospitalized in an acute care hospital for two or more days within 90 days before the current admission; 164 (23.4%) resided in a nursing home or long-term care facility; 136 (19.4%) had received intravenous antibiotic therapy, chemotherapy, or wound care within the past 30 days of the current infection; and 77 (11%) attended a hospital or hemodialysis clinic. We also included 934 CAP patients from the study hospitals for comparison. The flow diagram showing the number of cases and reasons for exclusion is shown in Figure S1. The demographic characteristics of the HCAP and CAP patients are shown in Table 1. Compared with the CAP patients, the HCAP patients were more likely to have a malignancy, renal insufficiency, diabetes, higher PSI score, and present with upper lung and/or bilateral lung involvement.

**Table 1:** Demographic profiles between hospitalized HCAP and CAP patients^a^.

	Overall, = 1635	Type of pneumonia		P value
		CAP, n = 934	HCAP, n = 701	
Mean age (SD)	72.8 (15.7)	72.5(16.6)	73.1 (14.5)	0.47
Male gender	1172 (71.7%)	674 (72.2%)	498 (71%)	0.62
Smoking habit	603 (36.9%)	329 (35.2%)	274 (39.1%)	0.11
Previous anti-TB treatment	82 (5%)	49 (5.2%)	33 (4.7%)	0.62
Comorbidities				
Malignancy	322 (19.7%)	79 (8.5%)	243 (34.7%)	<0.001
Renal insufficiency	199 (12.2%)	69 (7.4%)	130 (18.5%)	<0.001
Chronic liver disease	86 (5.3%)	41 (4.4%)	45 (6.4%)	0.07
Diabetes	443 (27.1%)	235 (25.2%)	208 (29.7%)	0.042
COPD	250 (15.3%)	154 (16.5%)	96 (13.7%)	0.12
Pneumonia severity				
PSI score	133.3 (34.5)	126.5 (31.7)	142.3 (36.0)	<0.001
CURB65 score	1.81 (1.07)	1.77 (1.05)	1.87 (1.10)	0.08
Chest film presentation				
Upper lung involvement	661 (40.4%)	339 (36.3%)	322 (45.9%)	<0.001
Bilateral lung involvement	703 (43%)	378 (40.5%)	325 (46.4%)	0.017
TB testing when admission	562 (34.4%)	375 (40.1%)	187 (26.7%)	<0.001
Pathogen in sputum culture (n = 744)				
Gram-positive bacteria	209 (28.1%)	131 (31.3%)	78 (24%)	0.08
Gram-negative bacteria	535 (71.9%)	288 (68.7%)	247 (76%)	0.06

a The data are presented as n (%) unless otherwise stated.

HCAP, healthcare-associated pneumonia; CAP, community acquired pneumonia; TB, tuberculosis; SD, standard deviation; COPD, chronic obstructive pulmonary disease; PSI, pneumonia severity index; CURB65, confusion, urea, respiratory rate, blood pressure, age 65.

The pathogens isolated from respiratory specimens of the included patients are shown in Table S1.

### Occurrence of concomitant PTB and clinical predictors

Seven HCAP patients had active PTB on admission, and a further 14 were diagnosed with PTB within 60 days after admission. Six CAP patients had active PTB on admission, and a further 19 were diagnosed with PTB within 60 days. The occurrence of concomitant PTB was comparable between the HCAP and CAP patients (3% vs. 2.7%, p = 0.73).

The demographic and clinical characteristics of the HCAP and CAP patients with or without concomitant PTB are shown in Table 2. In the HCAP group, patients with concomitant PTB were more likely to be older in age (79.6±13.4 vs. 72.9±14.5 years, p = 0.034), have a history of previous anti-TB treatment (23.8% vs. 4.1%, p<0.001), and have higher PSI scores (161.3±32.5 vs. 141.7±36, p = 0.017), as compared with those without concomitant PTB. In the CAP group, patients with concomitant PTB were more likely to have a previous history of anti-TB treatment (16% vs. 5%, p = 0.015), malignancy (20% vs. 8.1%, p = 0.036), and higher PSI scores (140.3±36.1 vs. 126.1±31.5, p = 0.033).

**Table 2:** Demographic profiles and clinical characteristics of CAP and HCAP patients with and without concomitant pulmonary tuberculosis^a^.

	Overall patient, n = 1635		P value	CAP patients, n = 934		P value	HCAP patients, n = 701		P value
	With TB, n = 46	Without TB, n = 1589		With TB, n = 25	Without TB, n = 909		With TB, n = 21	Without TB, n = 680	
Mean age (SD)	76.2 (15.3)	72.7 (15.7)	0.14	74.1 (16.8)	72.5 (16.6)	0.65	79.6 (13.4)	72.9 (14.5)	0.034
Male gender	38 (82.6%)	1134 (71.4%)	0.10	22 (88%)	652 (71.7%)	0.07	16 (76.2%)	482 (70.9%)	0.60
Smoking habit	20 (43.5%)	583 (36.7%)	0.35	12 (48%)	317 (34.9%)	0.18	8 (38.1%)	266 (39.1%)	0.93
Previous anti-TB treatment	9 (19.6%)	73 (4.6%)	<0.001	4 (16%)	45 (5%)	0.015	5 (23.8%)	28 (4.1%)	<0.001
Comorbidities									
Malignancy	14 (30.4%)	308 (19.4%)	0.06	5 (20%)	74 (8.1%)	0.036	9 (42.9%)	234 (34.4%)	0.42
Renal insufficiency	6 (13%)	193 (12.1%)	0.85	3 (12%)	66 (7.3%)	0.37	3 (14.3%)	127 (18.7%)	0.61
Chronic liver disease	1 (2.2%)	85 (5.3%)	0.34	1 (4%)	40 (4.4%)	0.92	0	45 (6.6%)	0.39
Diabetes	14 (30.4%)	429 (27%)	0.61	8 (32%)	227 (25%)	0.42	6 (28.6%)	202 (29.7%)	0.91
COPD	11 (23.9%)	239 (15%)	0.10	7 (28%)	147 (16.2%)	0.12	4 (19%)	92 (13.5%)	0.47
Pneumonia severity									
PSI score (SD)	149.9 (35.7)	132.8 (34.4)	0.001	140.3 (36.1)	126.1 (31.5)	0.033	161.3 (32.5)	141.7 (36.0)	0.017
CURB65 score (SD)	1.91 (1.05)	1.81 (1.08)	0.60	1.80 (1.00)	1.77 (1.05)	1.00	2.05 (1.12)	1.87 (1.10)	0.46
Chest film presentation									
Upper lung involvement	19 (41.3%)	642 (40.4%)	0.90	8 (32%)	331 (36.4%)	0.65	11 (52.4%)	311 (45.7%)	0.55
Bilateral lung involvement	24 (52.2%)	679 (42.7%)	0.20	12 (48%)	366 (40.3%)	0.44	12 (57.1%)	313 (46%)	0.31
Type of pneumonia			0.73			-			-
CAP	25 (54.3%)	905 (57%)		-	-		-	-	
HCAP	21 (45.7%)	684 (43%)		-	-		-	-	
TB testing on admission	25 (54.3%)	537 (33.8%)	0.004	16 (64%)	359 (39.5%)	0.014	9 (42.9%)	178 (26.2%)	0.089

a The data are presented as n (%) unless otherwise stated.

HCAP, healthcare-associated pneumonia; CAP, community acquired pneumonia; TB, tuberculosis; SD, standard deviation; COPD, chronic obstructive pulmonary disease; PSI, pneumonia severity index; CURB65, confusion, urea, respiratory rate, blood pressure, age 65.

The association between exposure to antibiotics and the occurrence of concomitant PTB is shown in Table 3. In both HCAP and CAP patients, the exposure to certain groups of antibiotics were comparable between patients with or without concomitant PTB.

**Table 3:** Relationships between antibiotics exposure and the occurrence of concomitant pulmonary tuberculosis in HCAP and CAP patients^a^.

Antibiotics exposure	CAP patients, n = 934		P value	HCAP patients, n = 701		P value
	With TB, n = 25	Without TB, n = 909		With TB, n = 21	Without TB, n = 680	
Penicillin						
Yes	15 (2.4%)	602 (97.6%)	0.52	16 (3.9%)	395 (96.1%)	0.10
No	10 (3.2%)	307 (96.8%)		5 (1.7%)	285 (98.3%)	
Cephalosporin						
Yes	6 (3.4%)	171 (96.6%)	0.51	3 (1.7%)	169 (98.3%)	0.27
No	19 (2.5%)	738 (97.5%)		18 (3.4%)	511 (96.6%)	
Macrolide						
Yes	5 (2.3%)	210 (97.7%)	0.72	1 (2.6%)	38 (97.4%)	1.00
No	20 (2.8%)	699 (97.2%)		20 (3%)	642 (97%)	
Carbapenem						
Yes	0	17 (100%)	1.00	0	27 (100%)	1.00
No	25 (2.7%)	892 (97.3%)		21 (3.1%)	653 (96.9%)	
Newer fluoroquinolones^b^						
Yes	4 (2%)	192 (98%)	0.47	4 (2.5%)	155 (97.5%)	0.69
No	21 (2.9%)	717 (97.1%)		17 (3.1%)	525 (96.1%)	

a The data are presented as n (%) unless otherwise stated.

b Included levofloxacin and moxifloxacin.

HCAP, healthcare-associated pneumonia; CAP, community acquired pneumonia; TB, tuberculosis.

In multivariate analysis, higher PSI scores (OR = 1.014, 95% CI: 1.002–1.026 in HCAP; OR: 1.013, 95% CI: 1.001–1.026 in CAP) and a previous history of anti-TB treatment (OR = 5.84, 95% CI: 2.29–20.37 in HCAP; OR: 3.33, 95% CI: 1.09–10.22 in CAP) were independent predictors of concomitant PTB in both HCAP and CAP patients (Table 4).

**Table 4:** Univariate and multivariate logistic regression analysis of predictors associated with concomitant pulmonary tuberculosis in hospitalized HCAP and CAP patients^a^.

	CAP patients				HCAP patients			
	Univariate		Multivariate		Univariate		Multivariate	
	OR (95% CI)	P value	OR (95% CI)	P value	OR (95% CI)	P value	OR (95% CI)	P value
Age	1.01 (0.98–1.03)	0.65			1.04 (1.003–1.086)	0.034		
Male gender	2.89 (0.86–9.74)	0.07			1.32 (0.48–3.64)	0.60		
Previous anti-TB treatment	3.66 (1.21–11.1)	0.015	3.33 (1.09–10.22)	0.035	7.28 (2.49–21.3)	<0.001	5.84 (2.29–20.37)	0.001
Malignancy	2.82 (1.03–7.72)	0.036			1.43 (0.59–3.44)	0.42		
Upper lobe involvement	0.82 (0.35–1.93)	0.65			1.31 (0.55–3.11)	0.55		
PSI score	1.01 (1.002–1.027)	0.033	1.013 (1.001–1.026)	0.038	1.015 (1.003–1.026)	0.015	1.014 (1.002–1.026)	0.023

a Univariate and multivariate OR are derived from logistic regression analysis with stepwise selection procedure.

HCAP, healthcare-associated pneumonia; CAP, community acquired pneumonia; TB, tuberculosis; OR, odds ratio; CI, confidence interval.

**Figure 1:**
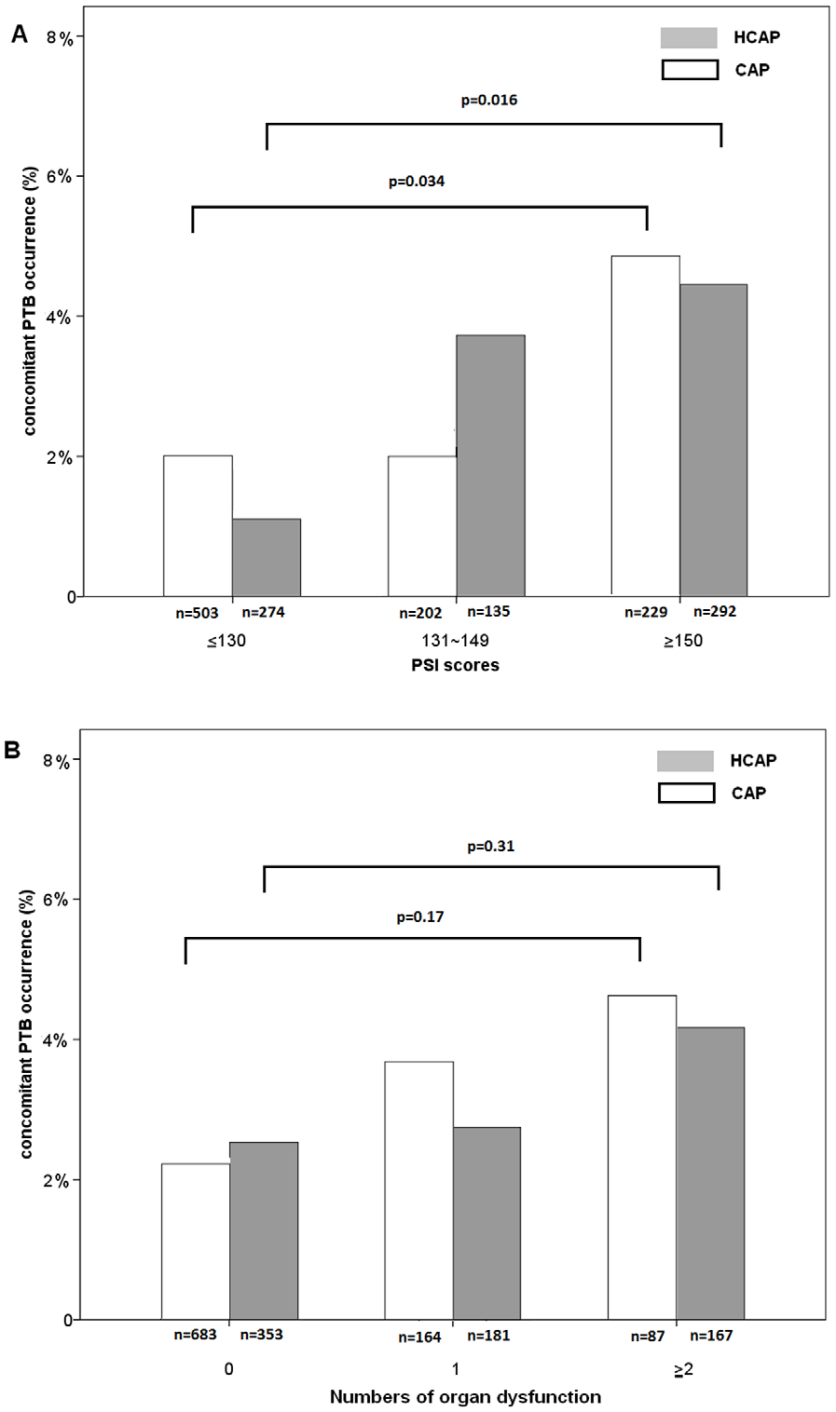
Occurrence of concomitant PTB among hospitalized HCAP and CAP patients with various severity. Patients are divided according to (A) PSI scores and (B) number of organ dysfunctions. HCAP, healthcare-associated pneumonia; CAP, community acquired pneumonia; PSI, pneumonia severity index; PTB, pulmonary tuberculosis.

The occurrence of concomitant PTB in HCAP and CAP patients stratified by the severity of PSI scores and the number of organ dysfunctions is shown in Figure 1. The occurrence of concomitant PTB increased as the PSI scores increased. Patients with PSI scores ≥150 had significantly more concomitant PTB than those with PSI scores ≤130, in both the HCAP (p = 0.016) and CAP (p = 0.034) groups. The occurrence of concomitant PTB also increased as the number of organ dysfunctions increased, although without statistical significance.

The demographic characteristics of the HCAP and CAP patients with or without concomitant PTB within 90 days and their clinical predictors are shown in Table S2 and Table S3.

### Impact of concomitant PTB in outcomes of the pneumonia patients

The treatment outcomes of the HCAP and CAP patients with or without concomitant PTB are shown in Table 5. In the HCAP group, patients with concomitant PTB had more acute respiratory failure within 48 hours of admission (47.6% vs. 22.6%, p = 0.008), higher ICU admission rate (61.9% vs. 35.7%, p = 0.014), and longer hospital stay (39.6±34.1 days vs. 23.7±27 days, p = 0.009) compared to those without concomitant PTB. In the CAP group, patients with concomitant PTB had a higher probability of ICU admission (44% vs. 19.7%, p = 0.003) and longer hospital stay (32±27.4 days vs. 19±20.3 days, p = 0.002).

**Table 5:** Impact of concomitant tuberculosis in treatment outcome of HCAP and CAP patients^a^.

	CAP patients, n = 934		P value	HCAP patients, n = 701		P value
	With TB, n = 25	Without TB, n = 909		With TB, n = 21	Without TB, n = 680	
Organs dysfunction within 48 hours						
Respiratory failure	3 (12%)	115 (12.7%)	0.92	10 (47.6%)	154 (22.6%)	0.008
Septic shock	4 (16%)	91 (10%)	0.33	5 (23.8%)	135 (19.9%)	0.66
Altered mental status	4 (16%)	66 (7.3%)	0.10	6 (28.6%)	145 (21.3%)	0.43
Renal dysfunction	2 (8%)	67 (7.4%)	0.91	1 (4.8%)	104 (15.3%)	0.18
Liver dysfunction	0	1 (0.1%)	1.00	0	3 (0.4%)	0.76
Coagulopathy	0	6 (0.7%)	1.00	1 (4.8%)	13 (1.9%)	0.36
Thrombocytopenia	2 (8%)	26 (2.9%)	0.14	1 (4.8%)	50 (7.4%)	0.65
ICU admission	11 (44%)	179 (19.7%)	0.003	13 (61.9%)	243 (35.7%)	0.014
Length of hospital stay (SD)						
All patients	32.0 (27.4)	19.0 (20.3)	0.002	39.6 (34.1)	23.7 (27.0)	0.009
Survivors	24.5 (17.9)	18.4 (19.7)	0.19	46.2 (42.6)	23.1 (23.9)	0.002
Non-survivors	56.1 (39.3)	23.4 (24.4)	0.003	32.4 (21.4)	25.7 (34.6)	0.54
28 days hospital free day (SD)	7.2 (8.9)	11.9 (8.8)	0.008	4.2 (7.5)	7.8 (8.5)	0.039
Mean Numbers of antibiotics used (SD)	1.74 (0.69)	1.61 (0.75)	0.41	1.29 (0.46)	1.37 (0.62)	0.52
Long-term ventilator dependent	1 (4%)	18 (2%)	0.48	1 (4.8%)	32 (4.7%)	0.99
Mortality						
30-day mortality	2 (8%)	73 (8%)	1.00	4 (19%)	133 (19.6%)	0.95
60-day mortality	3 (12%)	99 (10.9%)	0.86	7 (33.3%)	166 (24.4%)	0.35
In-hospital mortality	6 (24%)	106 (11.7%)	0.06	10 (47.6%)	179 (26.3%)	0.030

a The data are presented as n (%)unless otherwise stated.

HCAP, healthcare-associated pneumonia; CAP, community acquired pneumonia; TB, tuberculosis; SD, standard deviation; ICU, intensive care unit.

**Figure 2:**
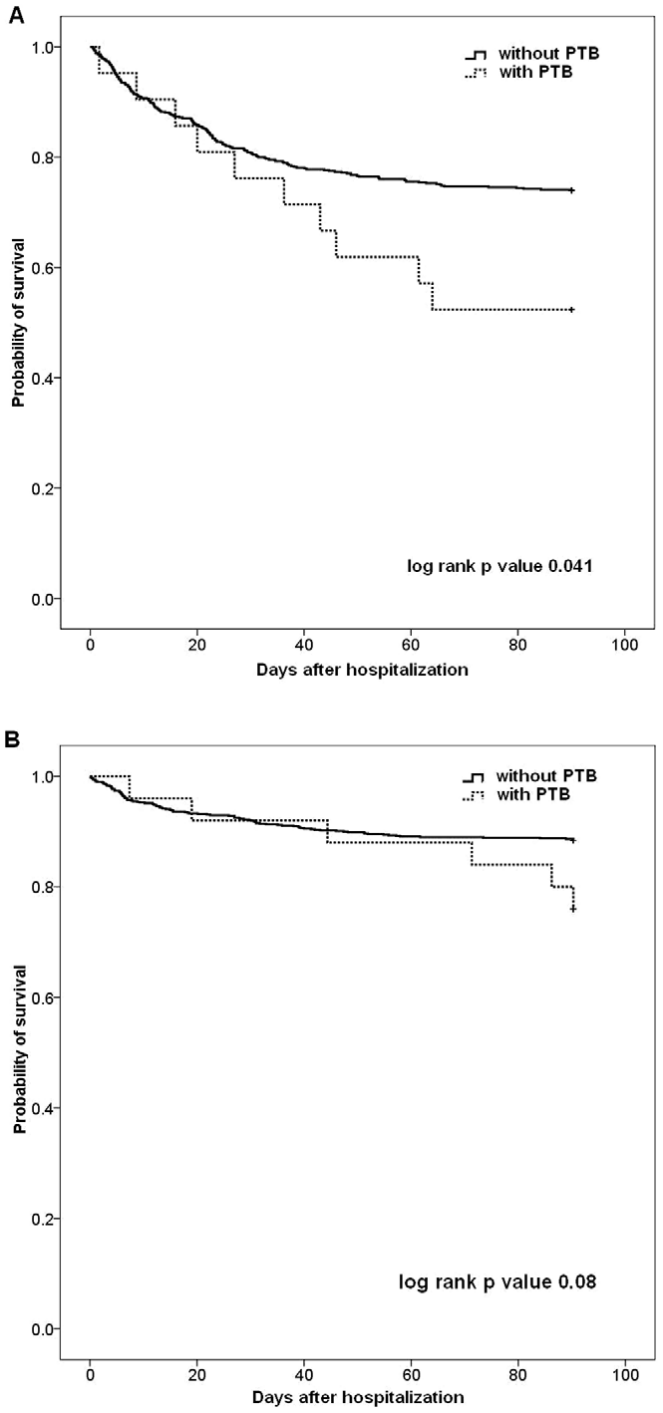
Kaplan-Meier survival curves of hospitalized pneumonia patients with or without concomitant PTB. Patients with (A) HCAP and (B) CAP were stratified by the presence or absence of concomitant PTB within 60 days. HCAP, healthcare-associated pneumonia; CAP, community acquired pneumonia; PTB, pulmonary tuberculosis.

Regarding mortality, the in-hospital mortality rate was higher in the HCAP patients with concomitant PTB (47.6% vs. 26.3%, p = 0.03) compared to those without PTB, although the 30-day and 60-day mortality rates were similar. The mortality rates were comparable in the CAP patients with or without concomitant PTB. Kaplan-Meier analysis of survival according to the presence or absence of concomitant PTB in the HCAP and CAP patients is shown in Figure 2. HCAP patients with concomitant PTB had significantly higher mortality (p = 0.041) compared to those without PTB. The survival curves overlapped between CAP patients with or without concomitant PTB.

Univariate and multivariate analysis of clinical predictors for in-hospital mortality in CAP and HCAP patients are shown in Table S4.

## Discussion

To the best of our knowledge, this is the first study to evaluate the predictors and outcomes of concomitant PTB in HCAP patients in a TB endemic area. We found that the occurrence of concomitant PTB in hospitalized HCAP patients was not rare and was comparable to those with CAP. A history of previous anti-TB treatment and higher PSI scores were independent predictors for the occurrence of PTB in both HCAP and CAP patients. We also found that HCAP patients with concomitant PTB were associated with worse outcomes of pneumonia, including more acute respiratory failure, higher ICU admission rate, longer hospital stay, and higher in-hospital mortality.

HCAP is a relatively new category of pneumonia that refers to pneumonias that occur prior to hospital admission in patients with specific risk factors [9]. In contrast to CAP, which is typically attributable to antibiotic-susceptible bacteria [14], patients with HCAP have a higher probability of being infected with antibiotic-resistant bacteria, and HCAP is usually associated with multiple comorbidities, higher disease severity and higher mortality [15]–[17]. There is a lack of an ideal scoring index for HCAP, however the applicability of PSI scores and CURB65 was reported in one recent study [18]. Compared with the CAP patients, the HCAP patients in our study had higher PSI scores and more advanced pulmonary involvement on chest radiographs. These patients also had a higher proportion of malignancies, renal insufficiency, and diabetes, which are well documented risk factors for PTB [19], [20]. Considering the multiple comorbidities and higher severity in HCAP patients, identifying the occurrence of concomitant PTB is an important issue, especially in TB endemic areas.

The association between CAP and PTB in TB endemic areas has been reported in previous studies [21], [22]. The latest guidelines for CAP also recommend TB studies as initial survey in TB endemic areas [10]. However, studies evaluating the association between HCAP and PTB are surprisingly rare. According to the latest HCAP guidelines, PTB should be considered only in non-responders to antibiotics treatment [9]. In our study, 3% of the hospitalized HCAP patients were documented to have concomitant PTB, which is comparable to the CAP patients. This indicates that PTB is not rare in HCAP patients in TB endemic areas, and that clinicians should keep PTB in mind when evaluating HCAP patients. Early diagnosis of concomitant PTB in HCAP is also important to prevent TB transmission in medical facilities.

The identification of clinical predictors associated with PTB in pneumonia patients helps in the early recognition of PTB. A prospective study performed in the Unites States indicated that typical CXR presentations, a history of previous TB, immigration status, weight loss and homelessness are independent predictors for PTB in patients presenting to the emergency department with a diagnosis of pneumonia [23]. In line with that report, a history of previous anti-TB treatment was an independent clinical predictor of PTB in the present study. We also found that higher PSI scores were independently associated with the presence of concomitant PTB in both HCAP and CAP patients. By comparison, age was not an independent predictor for concomitant PTB in multivariate analysis. One possible explanation is that the calculation of PSI scores already takes the factor of age into consideration. Another possible reason is that the correlation between age and higher disease severity may lessen the statistical significance of age in multivariate analysis. Furthermore, we clearly demonstrated that patients with PSI scores >130, which have been reported to have the highest risk of mortality [12], had a higher occurrence of PTB compared to those with PSI scores ≤130. Patients with higher disease severity may be associated with more comorbidities that increase the risk of PTB occurrence. Meanwhile, the presence of concomitant PTB may also complicate the disease course of pneumonia and increase disease severity. The higher disease severity and possible complications associated with anti-TB treatment may further worsen the treatment outcomes of these patients with pneumonia.

The impact of PTB on the outcomes of hospitalized pneumonia patients has been evaluated before with controversial results. Penner et al. reported higher mortality in patients with pneumonia and PTB requiring mechanical ventilation compared to those without PTB [24]. Another case control study reported comparable mortality rates between ICU admitted pneumonia patients with or without active PTB [25]. In our study, we found that HCAP patients with concomitant PTB were associated with higher in-hospital mortality. In multivariate analysis, the independent predictors for mortality of the HCAP patients were the presence of malignancy, higher PSI scores, higher CURB65 scores, and bilateral involvement in chest radiograms, but did not include the occurrence of concomitant PTB (data not shown). Our findings imply that the worse treatment outcomes of HCAP patients with PTB are more likely to result from the underlying comorbidities and higher disease severity, but not TB disease per se. Nevertheless, our findings still have important clinical implications. Given the fact that concomitant PTB is not rare in hospitalized HCAP patients, and that the higher disease severity in these patients lead to higher mortality, it is important that clinicians should consider PTB in HCAP patients with more comorbidities and higher severity. In addition, we also found that HCAP patients with concomitant PTB had more acute respiratory failure within 48 hours of admission, higher ICU admission rate, and longer hospital stay. Therefore, early diagnosis and early treatment of PTB may not only potentially reduce mortality, but also avoid TB transmission within healthcare facilities.

Our study has several limitations. This is a retrospective study and some clinical information may be incomplete. All of the participating hospitals are tertiary medical centers, and the enrolled patients were of older age with more comorbidities. Therefore, the incidence of concomitant PTB in these patients may be higher than that of the general population. Nevertheless, our findings can be applied to patients with more comorbidities. TB testing on admission was not routinely done in each patient. The occurrences of concomitant PTB may be underdiagnosed in HCAP group because of the lower TB testing rate on admission. However, our findings also clearly indicate the lack of awareness of PTB by clinicians, especially in HCAP patients. We did not stratify deaths as being due to either TB or HCAP in the study design because it is not always easy to clarify the causes of death in a retrospective study. All-cause mortality is more objective and applicable in clinical practice. Being a retrospective study, the MTB specimens from our patients could not be completely obtained, so drug susceptibility profiles were not demonstrated. Finally, this study was conducted in a TB endemic area with a low HIV prevalence rate, which may limit the application of our findings to non-HIV endemic areas.

In conclusion, our findings suggest that the occurrence of concomitant PTB in hospitalized HCAP patients in a TB endemic area is not rare and frequently overlooked by clinicians. Higher PSI scores and a history of previous anti-TB treatment were independent predictors for concomitant PTB in both hospitalized HCAP and CAP patients. Regarding treatment outcomes, hospitalized HCAP patients with concomitant PTB were associated with higher in-hospital mortality and more acute respiratory failure within 48 hours. These patients also had a higher ICU admission rate and longer hospital stay, which may lead to TB transmission if the diagnosis of PTB is delayed. Our results provide important evidence to remind clinicians to keep a high suspicion of and identify concomitant PTB early in hospitalized HCAP patients by examining the risk factors and pneumonia severity. Further prospective studies are warranted to elucidate this issue, especially in TB endemic areas.

## Supporting Information

Figure S1 **Study profile demonstrating the number of cases and reasons for exclusion.**
(TIF)

Table S1 **Pathogens isolated in respiratory specimens of CAP and HCAP patients^a^.**
(DOC)

Table S2 **Demographic profiles and clinical characteristics of CAP and HCAP patients with and without concomitant pulmonary tuberculosis within 90 days of admission^a^.**
(DOC)

Table S3 **Univariate and multivariate logistic regression analysis of predictors associated with concomitant pulmonary tuberculosis within 90 days of admission in hospitalized HCAP and CAP patients^a^.**
(DOC)

Table S4 **Univariate and multivariate logistic regression analysis of predictors associated with in-hospital mortality in hospitalized CAP and HCAP patients^a^.**
(DOC)
