# Supplementary figures and images for: Concomitant Pulmonary Tuberculosis in Hospitalized Healthcare-Associated Pneumonia in a Tuberculosis Endemic Area: A Multi-center Retrospective Study
Source: PLoS One. 2012 May 22;7(5):e36832. doi: 10.1371/journal.pone.0036832 (PMC3358294; doi:10.1371/journal.pone.0036832)

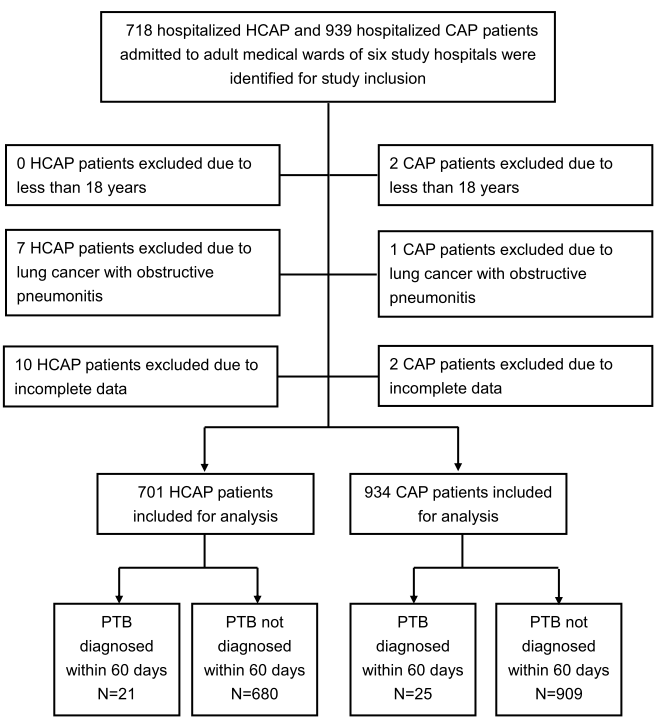

Supplement: Figure S1 — Study profile demonstrating the number of cases and reasons for exclusion. (TIF) [file pone.0036832.s001.tif]
