# Supplementary material for: Concomitant Pulmonary Tuberculosis in Hospitalized Healthcare-Associated Pneumonia in a Tuberculosis Endemic Area: A Multi-center Retrospective Study
Source: PLoS One. 2012 May 22;7(5):e36832. doi: 10.1371/journal.pone.0036832 (PMC3358294; doi:10.1371/journal.pone.0036832)
Supplement: Table S1 — Pathogens isolated in respiratory specimens of CAP and HCAP patientsa. (DOC) [file pone.0036832.s002.doc]

Table S1. Pathogens isolated in respiratory specimens of CAP and HCAP patients^a^

|  | Overall,n=1635 | Type of pneumonia | | P value |
| --- | --- | --- | --- | --- |
|  |  | CAP, n=934 | HCAP, n=701 |  |
| Gram-positive pathogen |  |  |  |  |
| Other *Streptococcus* spp. | 84 (5.1%) | 62 (6.6%) | 22 (3.1%) | 0.002 |
| MRSA | 68 (4.2%) | 32 (3.4%) | 36 (5.1%) | 0.09 |
| *Streptococcus pneumoniae* | 32 (2.6%) | 24 (2.6%) | 8 (1.1%) | 0.039 |
| MSSA | 20 (1.2%) | 9 (1.0%) | 11 (1.6%) | 0.27 |
| *Enterococcus* spp. | 2 (0.1%) | 1 (0.1%) | 1 (0.1%) | 1.00 |
| Others | 5 (0.3%) | 2 (0.2%) | 3 (0.4%) | 0.44 |
| Gram-negative pathogen |  |  |  |  |
| *Klebsiella* spp. | 199 (12.2%) | 109 (11.7%) | 90 (12.8%) | 0.47 |
| *Pseudomonas* spp. | 197 (12.0%) | 89 (9.5%) | 108 (15.4%) | <0.001 |
| *Acinetobacter* spp. | 54 (3.3%) | 31 (3.3%) | 23 (3.3%) | 0.97 |
| *Escherichia coli* | 53 (3.2%) | 32 (3.4%) | 21 (3.0%) | 0.63 |
| *Haemophilus influenza* | 50 (3.1%) | 33 (3.5%) | 17 (2.4%) | 0.20 |
| *Enterobacter* spp. | 43 (2.6%) | 17 (1.8%) | 26 (3.7%) | 0.018 |
| *Stenotrophmonas maltophilia* | 21 (1.3%) | 7 (0.7%) | 14 (2.0%) | 0.026 |
| *Serratia marcescens* | 20 (1.2%) | 8 (0.9%) | 12 (1.7%) | 0.12 |
| *Proteus mirabilis* | 16 (1.0%) | 5 (0.5%) | 11 (1.6%) | 0.035 |
| Others | 29 (1.8%) | 19 (2.0%) | 10 (1.4%) | 0.36 |

^a^ The data are presented as n (%) unless otherwise stated.

HCAP, healthcare-associated pneumonia; CAP, community acquired pneumonia; MRSA, methicillin-resistant *Staphylococcus aureus*; MSSA, methicillin-resistant *Staphylococcus aureus*
