# Supplementary material for: Concomitant Pulmonary Tuberculosis in Hospitalized Healthcare-Associated Pneumonia in a Tuberculosis Endemic Area: A Multi-center Retrospective Study
Source: PLoS One. 2012 May 22;7(5):e36832. doi: 10.1371/journal.pone.0036832 (PMC3358294; doi:10.1371/journal.pone.0036832)
Supplement: Table S2 — Demographic profiles and clinical characteristics of CAP and HCAP patients with and without concomitant pulmonary tuberculosis within 90 days of admissiona. (DOC) [file pone.0036832.s003.doc]

Table S2. Demographic profiles and clinical characteristics of CAP and HCAP patients with and without concomitant pulmonary tuberculosis within 90 days of admissiona

|  | CAP patients, n=934 | | P value | HCAP patients, n=701 | | P value |
| --- | --- | --- | --- | --- | --- | --- |
|  | With TB, n=29 | Without TB, n=905 |  | With TB, n=24 | Without TB, n=677 |  |
| Mean age (SD) | 75.7 (16.4) | 72.5 (16.6) | 0.30 | 79.7 (12.8) | 72.8 (14.5) | 0.022 |
| Male gender | 25 (86.2%) | 649 (71.7%) | 0.09 | 18 (75%) | 480 (70.9%) | 0.66 |
| Smoking habit | 15 (51.7%) | 314 (34.7%) | 0.06 | 9 (37.5%) | 265 (39.1%) | 0.87 |
| Previous anti-TB treatment | 5 (17.2%) | 44 (4.9%) | 0.015 | 6 (25%) | 27 (4%) | <0.001 |
| Comorbidities |  |  |  |  |  |  |
| Malignancy | 6 (20.7%) | 73 (8.1%) | 0.030 | 9 (37.5%) | 234 (34.6%) | 0.77 |
| Renal insufficiency | 3 (10.3%) | 66 (7.3%) | 0.47 | 3 (12.5%) | 127 (18.8%) | 0.60 |
| Chronic liver disease | 1 (3.4%) | 40 (4.4%) | 1.00 | 0 | 45 (6.6%) | 0.39 |
| Diabetes | 10 (34.5%) | 225 (24.9%) | 0.24 | 7 (29.2%) | 201 (29.7%) | 0.96 |
| COPD | 7 (24.1%) | 147 (16.2%) | 0.26 | 4 (16.7%) | 92 (13.6%) | 0.56 |
| Pneumonia severity |  |  |  |  |  |  |
| PSI score (SD) | 142.9 (34.9) | 126.1 (31.5) | 0.005 | 162.7 (33.1) | 141.6 (35.9) | 0.005 |
| CURB65 score (SD) | 1.86 (0.99) | 1.77 (1.05) | 0.63 | 2.04 (1.08) | 1.87 (1.10) | 0.45 |
| Chest film presentation |  |  |  |  |  |  |
| Upper lung involvement | 9 (31%) | 330 (36.5%) | 0.55 | 12 (50%) | 310 (45.8%) | 0.68 |
| Bilateral lung involvement | 16 (55.2%) | 362 (40%) | 0.10 | 14 (58.3%) | 311 (45.9%) | 0.23 |
| TB testing on admission | 18 (62.1%) | 357 (39.4%) | 0.014 | 9 (37.5%) | 178 (26.3%) | 0.22 |

^a^The data are presented as n (%) unless otherwise stated.

HCAP, healthcare-associated pneumonia; CAP, community acquired pneumonia; TB, tuberculosis; SD, standard deviation; COPD, chronic obstructive pulmonary disease; PSI, pneumonia severity index; CURB65, confusion, urea, respiratory rate, blood pressure, age 65
