# Supplementary material for: Concomitant Pulmonary Tuberculosis in Hospitalized Healthcare-Associated Pneumonia in a Tuberculosis Endemic Area: A Multi-center Retrospective Study
Source: PLoS One. 2012 May 22;7(5):e36832. doi: 10.1371/journal.pone.0036832 (PMC3358294; doi:10.1371/journal.pone.0036832)
Supplement: Table S3 — Univariate and multivariate logistic regression analysis of predictors associated with concomitant pulmonary tuberculosis within 90 days of admission in hospitalized HCAP and CAP patientsa. (DOC) [file pone.0036832.s004.doc]

Table S3. Univariate and multivariate logistic regression analysis of predictors associated with concomitant pulmonary tuberculosis within 90 days of admission in hospitalized HCAP and CAP patients^a^

|  | CAP patients | | | | HCAP patients | | | |
| --- | --- | --- | --- | --- | --- | --- | --- | --- |
|  | Univariate | | Multivariate | | Univariate | | Multivariate | |
|  | OR (95% CI) | P value | OR (95% CI) | P value | OR (95% CI) | P value | OR (95% CI) | P value |
| Age | 1.01 (0.99-1.04) | 0.30 |  |  | 1.04 (1.01-1.08) | 0.024 |  |  |
| Male gender | 2.47 (0.86-7.15) | 0.10 |  |  | 1.23 (0.48-3.15) | 0.66 |  |  |
| Smoking habit | 2.02 (0.96-4.23) | 0.06 |  |  | 0.93 (0.40-2.16) | 0.87 |  |  |
| Previous anti-TB treatment | 4.08 (1.49-11.2) | 0.006 | 3.10 (1.09-8.88) | 0.035 | 8.03 (2.95-21.83) | <0.001 | 6.69 (2.38-18.76) | <0.001 |
| Malignancy | 2.97 (1.17-7.53) | 0.022 |  |  | 1.14 (0.49-2.64) | 0.77 |  |  |
| Upper lobe involvement | 0.78 (0.35-1.74) | 0.55 |  |  | 1.18 (0.52-2.67) | 0.69 |  |  |
| PSI score | 1.01 (1.002-1.027) | 0.033 | 1.012 (0.998-1.025) | 0.084 | 1.015 (1.003-1.026) | 0.015 | 1.013 (1.001-1.023) | 0.039 |

^a^ Univariate and multivariate OR were derived from logistic regression analysis with stepwise selection procedure.

HCAP, healthcare-associated pneumonia; CAP, community acquired pneumonia; TB, tuberculosis; OR, odds ratio; CI, confidence interval; PSI, pneumonia severity index
