# Supplementary material for: Concomitant Pulmonary Tuberculosis in Hospitalized Healthcare-Associated Pneumonia in a Tuberculosis Endemic Area: A Multi-center Retrospective Study
Source: PLoS One. 2012 May 22;7(5):e36832. doi: 10.1371/journal.pone.0036832 (PMC3358294; doi:10.1371/journal.pone.0036832)
Supplement: Table S4 — Univariate and multivariate logistic regression analysis of predictors associated with in-hospital mortality in hospitalized CAP and HCAP patientsa. (DOC) [file pone.0036832.s005.doc]

Table S4. Univariate and multivariate logistic regression analysis of predictors associated with in-hospital mortality in hospitalized CAP and HCAP patients^a^

|  | CAP patients | | | | HCAP patients | | | |
| --- | --- | --- | --- | --- | --- | --- | --- | --- |
|  | Univariate | | Multivariate | | Univariate | | Multivariate | |
|  | OR (95% CI) | P value | OR (95% CI) | P value | OR (95% CI) | P value | OR (95% CI) | P value |
| Age | 1.02 (1.01-1.03) | 0.007 | 1.00 (0.98-1.02) | 0.94 | 1.00 (0.99-1.01) | 0.99 |  |  |
| Male gender | 1.57 (0.97-2.54) | 0.07 | 1.31 (0.79-2.19) | 0.30 | 1.19 (0.82-1.72) | 0.38 |  |  |
| Smoking habit | 0.86 (0.56-1.30) | 0.47 |  |  | 1.57 (1.12-2.21) | 0.009 | 1.13 (0.77-1.65) | 0.54 |
| Previous anti-TB treatment | 1.71 (0.81-3.62) | 0.16 |  |  | 1.02 (0.46-2.23) | 0.97 |  |  |
| Malignancy | 3.28 (1.91-5.61) | <0.001 | 2.20 (1.14-4.24) | 0.019 | 3.61 (2.55-5.11) | <0.001 | 3.86 (2.54-5.87) | <0.001 |
| Upper lobe involvement | 1.91 (1.28-2.83) | 0.001 | 1.57 (1.03-2.39) | 0.038 | 1.51 (1.08-2.11) | 0.016 | 1.21 (0.83-1.77) | 0.33 |
| Bilateral lung involvement | 2.90 (1.93-4.37) | <0.001 | 2.76 (1.80-4.24) | <0.001 | 1.98 (1.41-2.78) | <0.001 | 2.35 (1.59-3.46) | <0.001 |
| PSI score | 1.02 (1.012-1.026) | <0.001 | 1.01 (1.000-1.019) | 0.046 | 1.017 (1.012-1.022) | <0.001 | 1.007 (1.000-1.013) | 0.041 |
| CURB65 score | 1.58 (1.31-1.90) | <0.001 | 1.36 (1.07-1.72) | 0.011 | 1.42 (1.22-1.66) | <0.001 | 1.40 (1.15-1.71) | 0.001 |
| Concomitant PTB | 2.39 (0.94-6.12) | 0.07 | 1.82 (0.66-5.04) | 0.25 | 2.54 (1.06-6.09) | 0.036 | 2.05 (0.81-5.19) | 0.13 |

^a^ Univariate and multivariate OR were derived from logistic regression analysis with stepwise selection procedure.

HCAP, healthcare-associated pneumonia; CAP, community acquired pneumonia; TB, tuberculosis; OR, odds ratio; CI, confidence interval; PSI, pneumonia severity index; CURB65, confusion, urea, respiratory rate, blood pressure, age 65
